# BATF is Required for Treg Homeostasis and Stability to Prevent Autoimmune Pathology

**DOI:** 10.1002/advs.202206692

**Published:** 2023-08-16

**Authors:** Achia Khatun, Xiaopeng Wu, Fu Qi, Kexin Gai, Arjun Kharel, Matthew R. Kudek, Lisa Fraser, Ashley Ceicko, Moujtaba Y. Kasmani, Amber Majnik, Robert Burns, Yi‐Guang Chen, Nita Salzman, Elizabeth J. Taparowsky, Dayu Fang, Calvin B. Williams, Weiguo Cui

**Affiliations:** ^1^ Department of Microbiology and Immunology Medical College of Wisconsin Milwaukee WI 53226 USA; ^2^ Versiti Blood Research Institute Versiti Wisconsin Milwaukee WI 53226 USA; ^3^ Children's Mercy Hospital in Kansas City 2401 Gillham Rd Kansas City MO 64108 USA; ^4^ Department of Pathology Feinberg School of Medicine Northwestern University 303 E Chicago Ave Chicago IL 60611 USA; ^5^ Department of Pediatrics Medical College of Wisconsin 8701 Watertown Plank Road Milwaukee WI 53226 USA; ^6^ Max McGee National Research Center for Juvenile Diabetes Medical College of Wisconsin 8701 Watertown Plank Road Milwaukee WI 53226 USA; ^7^ Department of Biological Science Purdue University 610 Purdue Mall West Lafayette IN 47907 USA

**Keywords:** autoimmunity, BATF, Foxp3, Treg

## Abstract

Regulatory T (Treg) cells are inevitable to prevent deleterious immune responses to self and commensal microorganisms. Treg function requires continuous expression of the transcription factor (TF) FOXP3 and is divided into two major subsets: resting (rTregs) and activated (aTregs). Continuous T cell receptor (TCR) signaling plays a vital role in the differentiation of aTregs from their resting state, and in their immune homeostasis. The process by which Tregs differentiate, adapt tissue specificity, and maintain stable phenotypic expression at the transcriptional level is still inconclusivei. In this work, the role of BATF is investigated, which is induced in response to TCR stimulation in naïve T cells and during aTreg differentiation. Mice lacking BATF in Tregs developed multiorgan autoimmune pathology. As a transcriptional regulator, BATF is required for Treg differentiation, homeostasis, and stabilization of FOXP3 expression in different lymphoid and non‐lymphoid tissues. Epigenetically, BATF showed direct regulation of Treg‐specific genes involved in differentiation, maturation, and tissue accumulation. Most importantly, FOXP3 expression and Treg stability require continuous BATF expression in Tregs, as it regulates demethylation and accessibility of the CNS2 region of the *Foxp3* locus. Considering its role in Treg stability, BATF should be considered an important therapeutic target in autoimmune disease.

## Graphical abstract

Utilizing Treg‐specific BATF‐deficient mice, the authors unravel the crucial role of BATF in transcriptional regulation of genes that are specific to Treg differentiation, maturation, homeostasis, and tissue adaptability. Mechanistically, it is found that BATF plays a critical role in maintaining Treg stability and preserving FOXP3 expression by controlling the demethylation status and accessibility of the CNS2 region within the *Foxp3* locus.

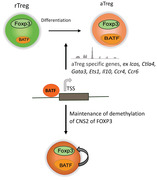

## 1 Introduction

Treg is a unique subset of CD4 T cells whose function is to inhibit deleterious, autoimmune responses while still enabling protective immune responses against foreign and infectious pathogens.^[^ ^1^, ^2^, ^3^ ^]^ A forkhead TF encoded on the X chromosome, FOXP3, has been identified as the major regulator of Treg programmatic development and function.^[^ ^4^, ^5^ ^]^ In humans, mutations in the *Foxp3* gene result in profound autoimmune pathology characterized by the immunodysregulation polyendocrinopathy enteropathy X‐linked (IPEX) syndrome.^[^ ^6^, ^7^ ^]^ In mice, a mutation in or deletion of the *Foxp3* gene similarly results in severe systemic autoimmunity and Treg deficiency known as the “Scurfy” phenotype.^[^ ^4^, ^8^ ^]^ Further, targeted deletion of *Foxp3* isolated to mature Tregs also results in the development of fatal autoimmune pathology.^[^ ^9^ ^]^ Collectively, these data indicate that Tregs are required for maintaining immune tolerance and that *Foxp3* maintains this phenotype through the entirety of the Treg lifecycle.

Due to the importance of *Foxp3* for maintaining Treg‐specific suppressive programs, the stability and regulation of *Foxp3* expression have been studied extensively.^[^ ^9^, ^10^, ^11^, ^12^, ^13^ ^]^ Demethylation of the conserved noncoding region 2 (CNS2) region of *Foxp3* is critical for stable *Foxp3* expression and is associated with the maintenance of an open chromatin structure that protects Treg identity and its suppressive program.^[^ ^10^, ^14^, ^15^ ^]^ Maintaining demethylation of the CNS2 region requires enzymatic activity of the TET (Ten eleven translocation) family of enzymes, which oppose DNA methyltransferase (DNMT) activity.^[^ ^16^, ^17^, ^18^ ^]^ In addition to epigenetic modification, transcriptional regulation of *Foxp3* is also mediated by the CNS2 region and its binding sites for numerous TFs including STAT5, NFAT, RUNX1/CBFB, CREB/ATF, Ets‐1, and GATA3, as well as a FOXP3 binding site to enable self‐regulation.^[^ ^10^, ^14^, ^15^, ^19^, ^20^, ^21^, ^22^, ^23^ ^]^

Thymically derived Tregs can be divided into two major subsets: resting rTregs and aTregs.^[^ ^24^, ^25^ ^]^ The resting subtype performs surveillance by circulating through blood and lymphatics and is characterized by CD62L^high^, CCR7^high^, and CD44^low^ surface expression of lymphoid homing molecules. In response to TCR‐mediated activation, rTregs undergo activation, develop a CD62L^low^, CD44^high^ phenotype, and express different chemokine receptors that enable migration to non‐lymphoid tissues.^[^ ^26^ ^]^

Both rTregs and aTregs possess non‐redundant homeostatic regulation and suppressive mechanisms.^[^ ^25^, ^27^, ^28^, ^29^, ^30^ ^]^ For example, rTregs express a high affinity IL‐2 receptor (CD25), which enables them to outcompete other T cells for IL‐2. Sequestration of IL‐2 is the major suppressive mechanism used by rTregs in the lymphoid tissues.^[^ ^3^, ^24^, ^26^ ^]^ In the activated state, aTregs express higher levels of Treg effector molecules like inducible T cell co‐stimulator (ICOS) and cytotoxic T‐cell associated antigen 4 (CTLA4) than rTregs. Sustained TCR signaling plays a vital role in the differentiation of aTregs and maintenance of peripheral immune tolerance and homeostasis.^[^ ^24^, ^31^, ^32^, ^33^ ^]^ aTregs are the dominant suppressive subset in the periphery, and expression of these surface molecules is important for their suppressive phenotype and survival.^[^ ^24^, ^26^, ^31^, ^33^, ^34^, ^35^ ^]^

Basic leucine zipper transcription factor ATF‐like (BATF), a TF from the basic leucine zipper (bZIP) or Activator Protein 1 (AP1) family is induced in response to TCR stimulation during aTreg differentiation.^[^ ^33^, ^36^, ^37^, ^38^, ^39^ ^]^ BATF is one of the most well‐studied TFs in terms of regulating the differentiation and function of multiple types of immune cells,^[^ ^38^, ^40^, ^41^, ^42^, ^43^, ^44^ ^]^ and has been identified as a pioneer TF in Th17 and Type 1 regulatory T cells (Tr1).^[^ ^45^, ^46^ ^]^ Consistent with this later role, others have shown that BATF is required to maintain chromatin accessibility at the precursor stage for ST2^+^ non‐lymphoid tissue‐resident Tregs.^[^ ^47^ ^]^

To date, the mechanism by which BATF regulates Treg differentiation, homeostasis, and stability in a cell‐specific manner is largely unknown. Furthermore, data supporting the role of BATF in regulating *Foxp3* expression and thus Treg stability are largely inconclusive. Herein, we report that Treg‐specific BATF deletion resulted in multiorgan immune‐mediated pathology with decreased survival. The absence of BATF in Tregs resulted in lowered accumulation or tissue adaption of Tregs in both lymphoid and non‐lymphoid tissues under both steady‐state and inflammatory conditions. In non‐lymphoid tissues, the absence of BATF in Tregs resulted in a transcriptomic profile that more closely resembled that of an effector T cell. The lack of BATF in Tregs also correlated with decreased *Foxp3* expression and Treg stability in both lymphoid and non‐lymphoid tissues over time. Mechanistically, BATF acts as a chromatin modifier, directly regulating chromatin accessibility of genes involved in the differentiation, suppressive function, proliferation, and stability of Tregs. Further, BATF also regulates chromatin accessibility at the CNS2 region of the *Foxp3* locus by maintaining demethylation of the CNS2 region, thereby stabilizing *Foxp3* expression.

## 2 Results

### 2.1 Treg‐Specific Batf Deletion Results in Multiorgan Autoimmune Dysregulation and Pathology

**Figure 1:**
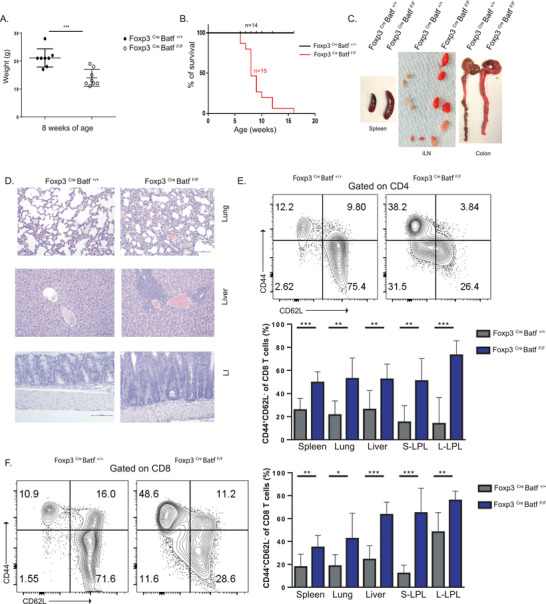
Treg‐specific BATF deletion results in homeostatic dysregulation with multiorgan autoimmune pathology. A) Body weight at 8 weeks of age of Treg‐specific BATF KO (*Foxp3* ^Cre^ *Batf* ^F/F^) mice and control mice *(Foxp3* ^Cre^ *Batf* ^+/+^). B) Survival curve of *Foxp3* ^Cre^ *Batf* ^F/F^ (*n* = 15, red line) compared to *Foxp3* ^Cre^ *Batf* ^+/+^ (*n* = 14, black line). C) Representative images showing development of splenomegaly, lymphadenopathy, and shorter, inflamed colons in *Foxp3* ^Cre^ *Batf* ^F/F^ mice compared to control mice around 8–10 weeks of age. D) H&E staining of tissue sections from lung, liver, and colon from *Foxp3* ^Cre^ *Batf* ^F/F^ mice compared to control mice (20× magnification). E,F) Frequency of activated E) CD4 and F) CD8 T cells in different tissues in *Foxp3* ^Cre^ *Batf* ^F/F^ mice compared to control around 8–10 weeks of age. S‐LPL, small intestine lamina propria. L‐LPL, large intestine lamina propria. All data are pooled from at least three individual repeats with a minimum of 3–5 mice per group. Data here are shown ± SEM. *p < 0.05, ^**^ *p* < 0.01, and ^***^ *p* < 0.001 (two tailed unpaired *t*‐test).

To determine the Treg‐specific role of BATF, *Foxp3^Cre^* mice were crossed with *Batf^F/F^* mice to generate mice with Treg‐specific BATF deficiency (*Foxp3^Cre^Batf^F/F^)*. *Foxp3^Cre^Batf^+/+^* mice were used as littermate controls (Figure S1A, Supporting Information). Expression of BATF in *Foxp3^Cre^Batf^F/F^* mice was significantly decreased in splenic and thymic Tregs as compared to controls (Figure S1B–D, Supporting Information). By 8 weeks of age, *Foxp3^Cre^Batf^F/F^* mice had a significant decrease in body weight (**Figure** **1**A), and most were moribund and spontaneously died shortly thereafter (Figure 1B). Gross splenomegaly, lymphadenopathy, and colitis observed in the absence of *Batf* (Figure 1C). This was accompanied by a significant increase in frequency of lymphocytes in the lymph nodes (Figure S2A, Supporting Information). Further, enhanced immune infiltrate and a loss of traditional architecture were histologically observed in multiple organs including lung, liver, and colon (Figure 1D). To determine if the inflammation observed in tissues was associated with homeostatic dysregulation, we analyzed the activation status of CD4 and CD8 T cells under steady‐state conditions. A significant increase in activated (CD44^high^ CD62L^low^) CD4 and CD8 T cells was observed in both lymphoid and non‐lymphoid organs in *Foxp3^Cre^Batf^F/F^* mice (Figure 1E,F). Functionally, the frequency of CD4 T cells producing IFN‐γ, IL‐17A, or TNF‐α and CD8 T cells producing IFN‐γ or TNF‐α were all significantly increased in the absence of BATF in Tregs (Figure S2B,C, Supporting Information). Among innate immune cell subsets, *Foxp3^Cre^Batf^F/F^* mice demonstrated a significant increase in both macrophages and neutrophils compared to controls (Figure S2D, Supporting Information). Taken together, the absence of BATF in Tregs led to multiorgan immune pathology and homeostatic dysregulation, likely due to activation and expansion of both adaptive and innate immune cells.

### 2.2 Altered Treg Fate and Activation Occur in BATF Deficiency

**Figure 2:**
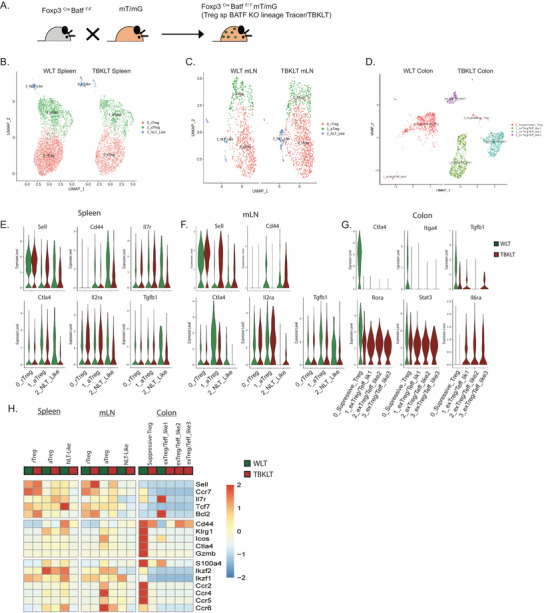
Altered Treg fate and activation occur in the absence of BATF function in Tregs. A) Schematic representation of the generation of Treg‐specific BATF KO lineage tracer, TBKLT (Foxp3^Cre^ Batf^F/F^ mT/mG) mice. B–D) UMAP plot showing clusters identified by variable gene expression from sorted GFP^+^ Tregs from B) spleen, C) mesenteric LN [mLN], and D) colon of TBKLT mice (Foxp3^Cre^ Batf ^F/F^) and WLT (Foxp3^Cre^ Batf^+/+^ mT/mG) mice at 7 weeks of age. E–G) Violin plots showing expression of Treg‐specific genes in E) spleen, F) mLN, and G) colon in WLT control (green) and TBKLT (red) mice. H) Heatmap showing Z scores for the average expression of Treg‐specific genes in each cluster between WLT (green) and TBKLT mice (red).

The severe increase in mortality seen in mice with Treg specific BATF deficiency led us to examine the role of BATF in Treg function and stability over time. To address this question, we generated a Treg‐specific *Batf* knockout lineage tracing (TBKLT) mouse model by crossing *Foxp3* ^Cre^ *Batf^F/F^* mice with mT/mG reporter mice (*Foxp3* ^Cre^ *Batf^F/F^* x mT/mG). The mT/mG mouse is a Cre‐recombinase driven reporter that expresses membrane‐targeted Tomato (mTomato or mT) prior to Cre excision, and membrane‐targeted green fluorescent protein (mGFP or mG) once Cre excision has occurred.^[^ ^48^ ^]^ A cross between *Foxp3* ^Cre^ *Batf* ^+/+^ mice and mT/mG mice served as *Batf* wildtype controls (WLT). In Tregs from both TBKLT and WLT mice, Tomato expression is permanently replaced by GFP once *Foxp3* is expressed (**Figure** **2**A). Phenotypically, TBKLT mice were found to be similar to *Foxp3* ^Cre^ *Batf^F/F^* mice (data not shown).

To understand the role of BATF as a transcriptional regulator of Treg subsets, single‐cell RNA sequencing (scRNA‐seq) was performed using GFP^+^ Treg cells sorted from spleens, mesenteric lymph nodes (mLN), and colons of WLT and TBKLT mice (Figure S3A, Supporting Information) at 7 weeks of age. Treg cells from spleen, mLN and colon of TBKLT mice showed significantly reduced *Batf* gene expression as compared to WLT (Figure S3B, Supporting Information). Following principal component analysis (PCA), cells from the secondary lymphoid organs (spleen and mLN) from both WLT and TBKLT mice were clustered into rTreg, aTreg, and non‐lymphoid tissue‐like regulatory T cells (NLT‐like Tregs) (Figure 2B,C). Cluster identities were assigned based on the differential expression of the lymphoid homing molecule and activation markers *Sell* (encodes CD62L), *Il7r, Ccr7, Cd44*, *Tcf7, Tnfrsf9*, and *Tnfrsf18* (Figure 2E,F,H; Figure S3C,D, Supporting Information) as reported previously.^[^ ^49^ ^]^ BATF‐deficient Treg cells were found to have lower expression of *Cd44* with higher expression of *Sell* and *Ccr7* compared to WLT (Figure 2E–G), a transcriptional profile consistent with less activation. Marginal reduction of other Treg‐specific genes involved in suppressive function (*Ctla4, Tgfb1, Icos*) and migration (*Ccr2, Ccr4, Ccr5*, and *Ccr6*) were also observed in the TBKLT group (Figure 2E,F,H).

In contrast to the markedly similar clustering and transcriptional profiles observed in Tregs from WLT and TBKLT secondary lymphoid organs, striking differences were observed in *Batf* sufficient and deficient colonic Tregs. In the WLT colon, nearly all Treg cells (99.8%) clustered into one major suppressive subset with enhanced expression of *Klrg1, Icos, Ctla4*, and *Gzmb* (Figure 2D,G,H; Figure S3E,F, Supporting Information). In comparison, altered Treg phenotypes were observed in TBKLT colons which clustered into 3 subsets following PCA, referred to as exTreg/Teff‐like1, exTreg/Teff‐like2, and exTreg/Teff‐like3. These Tregs had not only acquired a reduced expression of the Treg‐specific genes (*Foxp3*, *Ctla4*, and *Tgfb1), but* they had also developed Th17/Teff‐like features, with enhanced expression of Th17‐specific genes (*Rora, Stat3*, and *Il6ra)* (Figure 2D,G,H; Figure S3F, Supporting Information). Previous studies have shown that 40–60% of Tregs in the colon are inducible RORγt^+^ Th17‐like Tregs, and TGFβ1 regulates the Treg/Th17 cell fate differentiation.^[^ ^50^, ^51^ ^]^ In our studies, gene set module score analysis in the absence of BATF in colonic Treg cells demonstrated a significant reduction of Treg‐specific features and enhanced conventional T cell‐specific features (Figure S3G, Supporting Information). This finding was not due to differences in viability of lymphocytes recovered from intestinal lamina propria (Figure S4A, Supporting Information). Transcriptional differences in TBKLT and WLT Foxp3+ cells were also confirmed phenotypically. In TBKLT GFP^+^ Treg cells of the large intestine, a decrease in the expression of inducible costimulator (ICOS*)*, a protein associated with Treg differentiation and stability,^[^ ^52^ ^]^ and concomitant rise in expression of IL6 receptor (IL6ra) were identified (Figure S4B, Supporting Information). Taken together, these data suggest that BATF preserves the expression of Treg‐specific genes that regulate Treg activation and suppressive functions in secondary lymphoid organs. Further, BATF is also responsible for sustaining Treg lineage identity in peripheral, non‐lymphoid tissues like the colon.

### 2.3 BATF is Required for Treg Accumulation and Differentiation

**Figure 3:**
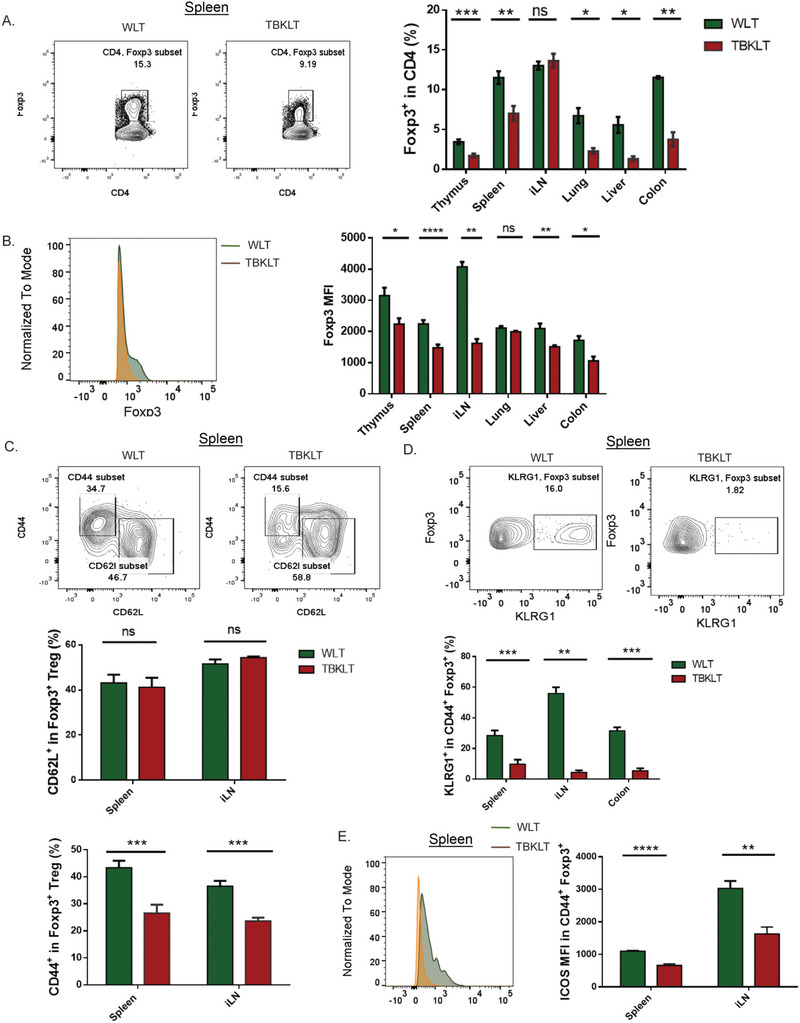
BATF is required for Treg accumulation and differentiation. A) Frequency of Foxp3^+^ Tregs in thymus, spleen, inguinal lymph nodes [iLN], lung, liver, and colon in TBKLT mice compared to control mice. Foxp3 expression is conferred by direct staining with and detection of intranuclear fluorophore‐conjugated antibody. B) Similar to A, but showing average expression of FOXP3 in CD4. C) Frequency of aTregs (CD62^low^ CD44^high^) and rTregs (CD62L^high^CD44^low^) in spleen and iLN of TBKLT mice and control mice. D) Frequency of KLRG1^+^ CD44^+^ FOXP3^+^ Tregs in spleen, iLN, and colon in TBKLT mice compared to WLT control mice. E) Average expression of ICOS in CD44^+^ FOXP3^+^ Tregs in TBKLT mice and control mice. All data are from mice between 5 to 11 weeks of age and are pooled from at least three independent experiments with 2–3 mice per group. Data here are shown ± SEM. *p < 0.05, ^**^ *p* < 0.01, and ^***^ *p* < 0.001 (two tailed unpaired *t*‐test).

We next sought to determine if the homeostatic dysregulation of effector T cells (Figure 1E,F) resulted from either a decreased prevalence of Treg cells, diminished suppressive function, or both. Indeed, in both non‐lymphoid and secondary lymphoid organs of TBKLT mice, the frequency of FOXP3^+^ Treg cells and the per‐cell expression of FOXP3 were both reduced relative to WLT‐derived Treg cells (**Figure** **3**A,B). We also found that while the frequency of rTregs between TBKLT and WLT mice was unchanged in the secondary lymphoid tissues assayed, the frequency of aTregs (CD44^high^ CD62L^low^) was significantly reduced (Figure 3C). This suggests that Treg‐produced BATF is required for Treg accumulation and *Foxp3* expression and serves to sustain tissue‐specific homeostatic regulation.

In line with this, reduced inducible Treg (iTreg) cell frequency and FOXP3 expression were observed in TBKLT T cells following in vitro culture of naïve CD4 T cells under Treg skewing culture conditions (Figure S5A–C, Supporting Information). Comparable *Ki67* expression was observed between the two groups of iTregs (Figure S5D, Supporting Information), suggesting that reduced iTreg differentiation was not due to impaired proliferation.

To further validate our scRNA‐seq findings, we analyzed Treg cells from TBKLT and WLT mice for functional correlation to the observed transcriptomics. As expected, a significant reduction in the frequency of KLRG1^+^ terminally differentiated aTregs was found in the TBKLT spleen, inguinal lymph nodes (iLN), and colon (Figure 3D). This corroborated the reduced transcription of *Klrg1* observed in the aTreg and suppressive‐Treg subsets observed in the scRNA‐seq data (Figure 2H). Similarly, we examined these TBKLT and WLT Treg cells for ICOS expression, which is critical for aTreg survival and suppressive function.^[^ ^26^, ^52^ ^]^ Like the transcriptional profile observed for *Icos* in the aTreg subsets (Figure 2H), ICOS surface expression was significantly decreased in BATF‐deficient Tregs of the spleen and iLN (Figure 3E). Overall, our data indicate that BATF regulates and is required for Treg activation, maturation and differentiation, consistent with previous studies.^[^ ^36^, ^47^, ^53^ ^]^

### 2.4 BATF is Intrinsically Required for Treg Homeostasis and Competitive Fitness

**Figure 4:**
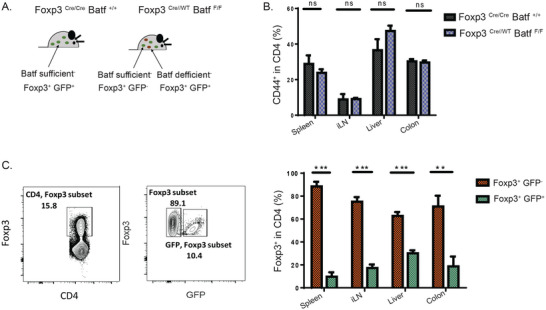
BATF is intrinsically required for Treg homeostasis and competitive fitness. A) Schematic representation of TBKLT mosaic (Foxp3^Cre/WT^ Batf^F/F^) mice compared to control (Foxp3^Cre/Cre^ Batf^+/+^) mice. B) Frequency of activated CD4 T cells in TBKLT mosaic mice compared to control mice. C) Frequency of Foxp3^+^ GFP^−^ (BATF sufficient) and Foxp3^+^ GFP^+^ (BATF deficient) Tregs in spleen, iLN, liver, and colons of female TBKLT mosaic mice. FOXP^+^ expression is conferred by direct staining with and detection of intranuclear fluorophore‐conjugated antibody. Data are from pooled two independent experiments using female TBKLT mosaic and control mice, around 3–4 months of age, and 2 mice per group. Data here are shown ± SEM. *p < 0.05, ^**^ *p* < 0.01, and ^***^ *p* < 0.001 (two tailed unpaired *t*‐test).

Based on observations made in previous studies, we hypothesized that the decreased Treg frequency in TBKLT mice may be due to a consequence of ongoing inflammation and the presence of pro‐inflammatory cytokines.^[^ ^54^, ^55^, ^56^ ^]^ To evaluate this possibility and understand the role of BATF in Tregs in a cell‐intrinsic manner, we generated BATF chimeric (TBKLT mosaic) female mice, homozygous for the *Batf^F^* allele (F/F) and heterozygous for *Foxp3* ^Cre^ allele (Cre/WT) (**Figure** **4**A). As *Foxp3* is an X‐linked gene, the random inactivation of one X chromosome in cells of female mouse results in mosaic expression of BATF sufficient (Foxp3^+^GFP^−^) and BATF deficient (Foxp3^+^GFP^+^) cells in the Treg compartment of each mouse. TBKLT mosaic female mice were healthy and did not develop any observed overt disease out to 16 weeks of age. Supporting this, no significant difference in the frequency of activated CD4 T cells was observed between TBKLT mosaic female mice and their Foxp3^Cre/Cre^ Batf^+/+^ littermate controls, excluding the possibility of homeostatic dysregulation in these mice (Figure 4B). Matched comparisons of BATF‐sufficient (Foxp3^+^GFP^−^) and ‐deficient (Foxp3^+^ GFP^+^) Tregs were performed within each TBKLT mosaic mice in lymphoid and non‐lymphoid tissues (Figure 4C). Significant decreases in BATF‐deficient Treg frequencies were observed in all tissues examined. To ensure that these differences were not due to degradation of cell surface GFP, we confirmed by flow cytometry that intracellular staining for FOXP3 did not result in an appreciable difference in the frequency of GFP^+^ cells (data not shown). These findings suggest that BATF sufficiency enables the competitive fitness of Tregs in a cell‐intrinsic manner under homeostatic conditions.

### 2.5 BATF Regulates Chromatin Accessibility of Treg‐Specific Genes

**Figure 5:**
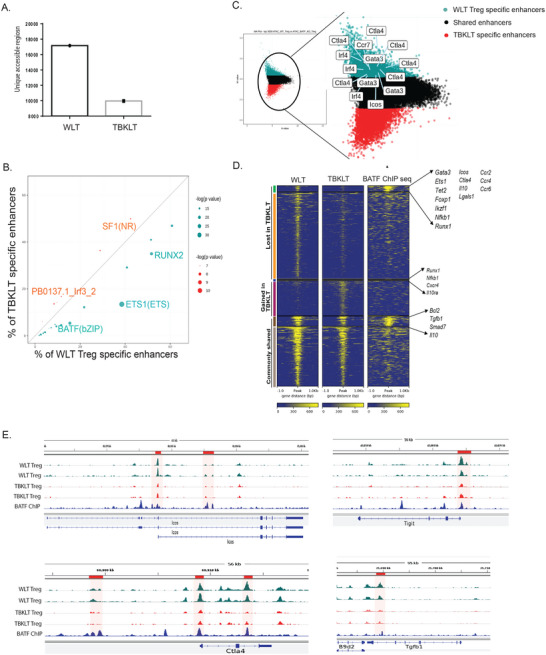
BATF regulates chromatin accessibility of Treg‐specific genes. A) Bar plot showing the number of uniquely accessible enhancer regions in TBKLT mice compared to WLT control mice. B) Dot plot showing top enriched motifs in the uniquely accessible top 5000 enhancer regions in WLT and TBKLT mice. **C)** MA Plot showing top 5000 unique enhancer regions and associated genes accessible in Tregs from either WLT mice (cyan) or TBKLT mice (red). D) Heatmap with top 5000 uniquely accessible enhancer regions from either WLT (left), TBKLT mice (middle), and published BATF ChIP‐seq data (right) in Tregs. Green and orange bars indicate enhancer regions bound and not bound by BATF, respectively, accessible in WLT mice but lost in TBKLT mice. Blue and purple bars indicate enhancer regions bound and not bound by BATF, respectively, not accessible in WLT mice but accessible in TBKLT mice. Gray and light gray bars indicate enhancer regions bound and not bound by BATF, respectively, with similar accessibility in WLT and TBKLT mice. Representative genes associated with the enhancer regions are annotated by arrows to the right of the panel. E) Representative tracks of WLT and TBKLT Treg ATAC‐seq data and published BATF ChIP‐seq data in Treg in enhancer regions of Treg‐specific genes like *Icos, Ctla4, Tigit*, and *Tgfb1* in WT versus TBKLT (orange bar). Figure A–D data is representative of two independent experiments performed on mice aged 7 weeks.

As a well‐described pioneer TF and chromatin modifier in Th17, Tr1, and CD8 T cells,^[^ ^39^, ^45^, ^46^, ^57^ ^]^ we sought to determine if BATF played a similar role in Treg cells. To do so, we utilized the Assay for Transposase Accessible Chromatin with sequencing (ATAC‐seq) to investigate the accessibility of Treg‐specific genes in BATF‐sufficient and BATF‐deficient Tregs. ATAC‐seq was performed on 5 × 10^5^ GFP^+^ Tregs sorted from both TBKLT and WLT mice (Figure S6A, Supporting Information). The initial analysis demonstrated that WLT mice had nearly three times as many uniquely accessible regions compared to TBKLT mice (**Figure** **5**A; Figure S6B, Supporting Information). Subsequent motif analysis of these regions showed enrichment of BATF (bZIP), ETS1, and RUNX family TF motifs in WLT mice compared to TBKLT mice (Figure 5B; Figure S6C, Supporting Information). Gene association analysis of these uniquely accessible regions showed that Treg‐specific genes like *Ctla4, Icos, Gata3*, and *Irf4* were only identified in WLT, but not in TBKLT (Figure 5C). Supporting this, analysis of WLT and TBKLT GFP^+^ Tregs showed a total of 503 regions with significantly different accessibility. Of these, 498 regions (99%) were uniquely open in WLT and lost in TBKLT (Figure S6D, Supporting Information). Gene annotation of these regions lost in TBKLT mice showed an association with TFs including *Ets1, Ikzf1*, and *Nfkb1*, immune suppressive molecules like *Ctla4, Tgfb1, Lgals1*, and *Gzmb*, and genes involved in chemotaxis and the cell cycle. Pathway enrichment analysis of the accessible enhancer regions in WLT GFP^+^ Tregs showed associations with multiple pathways associated with leukocyte regulation in WLT mice in line with normal Treg function. Similar pathway associations were notably absent in analyses performed on enhancer accessible regions in BATF‐deficient, TBKLT GFP^+^ Tregs (Figure S6E,F, Supporting Information).

To understand direct regulation by BATF, ATAC‐seq data were combined with published BATF Chromatin Immunoprecipitation with sequencing (ChIP‐seq) data in Tregs.^[^ ^53^ ^]^ Treg‐specific genes bound by BATF included those involved in Treg homeostasis (*Gata3, Ets1, Ikzf1, Bach2, Foxp1*, and *Nfkb1*), suppressive function (*Icos, Ctla4, Il10*, and *Tgfb1*), migration (*Ccr2, Ccr4*, and *Ccr6*) and cell cycle (*Cdk13, Cdk14*, and *Cdk16*). Many of these genes showed reduced accessibility in their enhancer regions in TBKLT GFP^+^ Tregs relative to their WLT counterparts (Figure 5D). Further, several genes related to Treg suppressive function lost enhancer accessibility in TBKLT Tregs, including *Ctla4, Icos, Tigit*, and *Tgfb1* (Figure 5E). Taken together, these data enforce the notion that BATF directly regulates the accessibility of canonical genes known to be essential for Treg homeostasis, tissue accumulation, and function.

### 2.6 Treg Stability and FOXP3 Expression Require BATF Function in Tregs

**Figure 6:**
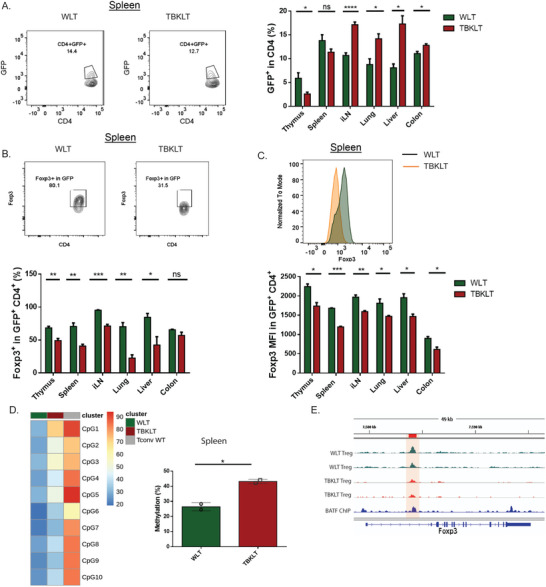
Treg stability and FOXP3 expression require BATF function in Tregs. A) Frequency of GFP^+^ Tregs in thymus, spleen, iLN, lungs, liver, and colon of TBKLT and WLT control mice aged 10–12 weeks. B,C) Similar to A, but showing frequency of FOXP3^+^ cells in GFP^+^ Tregs and MFI of FOXP3. D) Heatmap and bar plot showing percentage of methylation at the CNS2 region of the *Foxp3* locus in sorted GFP^+^ Tregs from spleens of WLT and TBKLT mice at 7 weeks of age. WT CD4 T cells (Tconv WT) were used as a negative control. E) Representative tracks of ATAC‐seq data and published BATF ChIP‐seq data in Tregs showing binding of BATF at the CNS2 region of the *Foxp3* locus. Data in B–D are pooled from two independent experiments with 1–2 mice per group. Data here are shown ± SEM. *p < 0.05, ^**^ *p* < 0.01, and ^***^ *p* < 0.001 (two tailed unpaired *t*‐test).

Continuous expression of *Foxp3* is required for Treg‐mediated immune suppression and homeostasis.^[^ ^9^ ^]^ Persistent demethylation of the CNS2 region in the *Foxp3* locus allows binding of FOXP3 and co‐factors including NFAT, RUNX1/CBFB, CREB/ATF, ETS1, GATA3 and STAT5, which reinforce *Foxp3* expression.^[^ ^10^, ^23^ ^]^ Decrease in FOXP3 expression in different tissues from 5 to 11 weeks of age (Figure 3B) led us to question whether BATF is required for the stability of *Foxp3* expression in Tregs. To explore this, we assessed differences in intracellular staining levels for FOXP3 in GFP^+^ TBKLT and WLT Tregs to approximate the level of *Foxp3* expression. As such, differences in frequency of FOXP3 expression between the GFP^+^ TBKLT and WLT Treg cells would reflect differential *Foxp3* expression between the two, thereby allowing us to interrogate the requirement for BATF in stabilizing *Foxp3* expression over time. Subsequently, we observed a decrease in the frequency of GFP^+^ Tregs in the TBKLT thymus, but an increase in frequency of GFP^+^ Treg cells in the TBKLT iLN, lung, liver, and colon (**Figure** **6**A). Despite this, the frequency of GFP^+^ Treg cells expressing FOXP3^+^ was significantly decreased in both lymphoid and non‐lymphoid tissues analyzed from TBKLT mice when compared to their WLT controls (Figure 6B). In line with these results, average FOXP3 expression assessed by mean fluorescence intensity was also significantly decreased in TBKLT GFP^+^ Tregs in lymphoid and non‐lymphoid tissues (Figure 6C), suggesting unstable *Foxp3* expression in BATF deficient Tregs.

Supporting this, we observed increased methylation of the CNS2 region of the *Foxp3* locus in BATF‐deficient Tregs, correlating with reduced chromatin accessibility (Figure 6D,E). This suggests that a direct role of BATF is to stabilize *Foxp3* expression by preventing methylation of the CNS2 region. Additionally, we observed reduced accessibility of BATF binding *Gata3* and *Ets1* enhancer regions in TBKLT Tregs (Figure S7A,B, Supporting Information). As GATA3 and ETS1 are involved in stabilizing *Foxp3* expression by binding to the CNS2 region as part of a complex with FOXP3,^[^ ^15^, ^22^ ^]^ BATF may also indirectly stabilize expression of *Foxp3*.

To further validate the proposed role for BATF in stabilizing *Foxp3* expression, we induced deletion of BATF *ex vivo* with tamoxifen treatment of sorted CD4^+^ CD25^high^ Tregs from ER^Cre^ *Batf ^F/F^* mT/mG mice (Figure S8A,B, Supporting Information).^[^ ^58^ ^]^ Following the administration of tamoxifen, no significant difference in frequency of CD4^+^CD25^high^ Tregs was observed between the BATF‐deplete and BATF‐replete cells. (Figure S8C, Supporting Information). Tamoxifen treatment increased the frequency of GFP^+^ CD4^+^ CD25^+^ cells from Cre excision of membrane‐targeted tomato (mT or mTomato) protein compared to control. (Figure S8D, Supporting Information). This confirmed the efficacy of tamoxifen treatment as reported before.^[^ ^59^, ^60^ ^]^ As deletion of BATF resulted in a significant reduction of FOXP3 expression (Figure S8E,F, Supporting Information), this again confirmed a role for BATF in sustaining *Foxp3* expression, further supporting our in vivo data from lineage tracer mice.

## 3 Discussion

Here we have demonstrated the critical role of BATF in the maintenance of physiologic Treg function. We have shown that in the absence of BATF, multiorgan autoimmune pathology occurs, with homeostatic dysregulation involving both adaptive and innate immune cell types. Further, we have described how BATF is required for Treg activation and differentiation and is essential for the maintenance of Treg lineage specificity, particularly in non‐lymphoid organs like the colon. Our data suggest that there are two mechanisms by which BATF stabilizes *Foxp3* expression over time. One is through regulation of the maintenance of demethylation, and therefore accessibility, of the CNS2 region of the *Foxp3* locus, a key transcription factor for Treg function. The second mechanism is through regulation of other genes involved in Treg stability. In total, we have demonstrated that BATF function in Treg cells is required for Treg‐mediated immune homeostasis and that the loss of BATF contributes to autoimmune pathology.

TCR‐dependent differentiation, tissue adaptation, and functional maturation of Tregs are integral parts of Treg‐mediated homeostatic regulation and immune tolerance. Previous studies have shown that transcriptional regulators involved in effector T cell differentiation are also involved in the regulation of Treg function in inactive and activated states.^[^ ^61^ ^]^ In line with this, our study focused on BATF, which is a pioneer TF that acts as a transcriptional regulator and chromatin modifier in many types of immune cells.^[^ ^38^, ^40^, ^43^, ^45^, ^62^ ^]^ In our study, the focus was to understand the role of BATF in different aspects of Treg biology including differentiation, function, and maintenance. Here we used TBKLT mice to show that conditional deletion of BATF in Tregs impaired three key aspects of Treg biology: 1) suppressive function and homeostatic regulation, 2) differentiation/maturation and tissue adaptation, and 3) stability of Foxp3 expression and maintenance of Treg over time. Mechanistically, we found BATF was crucial to regulate Treg‐specific transcriptional programming and maintain demethylation of the CNS2 region of Foxp3 gene.

Tregs exist in a resting state (rTregs), which differentiate into an activated state (aTreg) in secondary lymphoid tissues in response to TCR signaling, IL‐2, and cytokine stimulation.^[^ ^24^, ^26^ ^]^ BATF, a well‐studied TF regulating differentiation of multiple types of immune cells, has been found to be induced in the generation of aTregs.^[^ ^36^, ^47^ ^]^ We observed that selective deletion of BATF in Tregs resulted in impaired Treg differentiation, tissue adaptation, and alteration of Treg fate, most visibly in non‐lymphoid tissues like the colon. It is known that ROR‐γt^+^ iTregs that produce and are regulated by TGFβ1 are the major Treg subset to maintain immune homeostasis in the colon.^[^ ^50^, ^51^ ^]^ Here we showed that when absent of BATF, Tregs from the colon adopted a T effector‐like phenotype with reduced FOXP3 and TGFβ1 expression. This suggests a role for BATF in regulating Treg lineage specificity in peripheral tissues and a possible role in regulating iTreg differentiation, as further supported by our in vitro iTreg induction results.

Inflammation can affect in vivo Treg tissue adaptation and stability of FOXP3 expression.^[^ ^54^, ^55^, ^56^ ^]^ Using TBKLT mosaic female mice, we found significantly reduced frequencies of FOXP3‐expressing Tregs when they were BATF‐deficient. The Treg‐specific transcriptional program is regulated by many TFs including FOXP3, and epigenetic regulation of FOXP3 expression has been examined in a recent study which found that ETS and bZIP family TFs control the accessibility of regulatory elements bound by *Foxp3* in Treg‐specific genes, independent of FOXP3 expression.^[^ ^63^ ^]^ Supporting this, we found that BATF, a member of the bZIP family, regulates accessibility of Treg‐specific genes. Differential accessibility analysis showed a loss of 498 enhancer regions in the absence of BATF in Tregs. From the combined analysis of ATAC‐seq with published BATF ChIP‐seq data on Tregs,^[^ ^53^ ^]^ BATF was found to directly regulate the accessibility of the enhancer regions of genes involved in Treg function, migration to inflammatory sites, and the cell cycle. Together these data demonstrate that BATF directly maintains the core Treg transcriptome, and therefore, Treg suppressive function, by regulating chromatin accessibility to facilitate transcription.

Maintenance of Treg function requires the continuous expression of FOXP3 in Tregs.^[^ ^9^ ^]^ FOXP3, along with cofactors CREB/ATF, NFκB, ETS1, RUNX1, c‐Rel, STAT5, and GATA3, bind to the demethylated CNS2 region of the *Foxp3* locus to stabilize its own expression.^[^ ^10^, ^14^, ^15^, ^19^, ^20^, ^21^, ^23^ ^]^ As we observed both reduced Treg frequency and lower levels of FOXP3 in TBKLT mice, our data indicate a central role for BATF in maintaining a level of *Foxp3* expression that is critical for Treg stability. Furthermore, in the absence of BATF, FOXP3 was significantly reduced in GFP^+^ Tregs in different lymphoid and non‐lymphoid tissues, suggesting a role for BATF in stabilizing *Foxp3* expression in both thymically derived Tregs and Tregs generated in periphery.

One caveat of our approach using GFP^+^ Tregs to trace *Foxp3* expression was the inclusion of both continuous thymic input and cells that transiently upregulated FOXP3/YFP inside the GFP^+^ Treg compartment. Distinguishing YFP^+^ (also FOXP3^+^) and GFP^+^ (which may or may not be expressing FOXP3) could help to interpret the Treg cell state used in different experiments. However, this was not feasible in our study due to an inability to separately identify YFP and GFP by flow cytometry. We attempted to circumvent this and examine the role of BATF in a setting devoid of inflammation by inducing deletion of BATF from mature Tregs in vitro using tamoxifen‐treated Treg cells from ER^Cre^ BATF^F/F^ mT/mG mice. A significant reduction in FOXP3 was observed following deletion of BATF, suggesting BATF is required to maintain *Foxp3* in Tregs under homeostatic conditions. These data reinforce our interpretation that the decrease in FOXP3^+^ Treg frequency and the average cellular FOXP3 expression seen in GFP^+^ Tregs in vivo is due to instability of *Foxp3* expression in BATF‐deficient Tregs.

As there is reduced accessibility of regions normally bound by BATF in the CNS2 region of the *Foxp3* locus in TBKLT mice, the data support a BATF‐mediated regulatory mechanism of stabilizing *Foxp3* expression. This is further supported by our observation of increased methylation at the CNS2 region of *Foxp3* in the absence of BATF. While BATF facilitates *Aicda* expression through recruitment of TET demethylase enzymes in B cells,^[^ ^64^ ^]^ our scRNA sequencing data did not identify an appreciable difference in expression of TET or DNMT family gene transcription (data not shown), suggesting that BATF may indirectly regulate this process in Tregs. Future studies are needed to establish this link more definitively. However, other genes involved in regulating *Foxp3* stability, including *Gata3* and *Ets1*,^[^ ^10^, ^65^, ^66^ ^]^ had reduced chromatin accessibility in TBKLT mice in regions normally bound by BATF. This suggests that BATF may also regulate the expression of *Gata3* and *Ets1*, which thereby also indirectly regulates *Foxp3*. Future investigations are also warranted to unravel these mechanisms.

Ensuring the stability of Tregs is one of the major challenges for the therapeutic usage of Tregs in the treatment of autoimmune disease and prevention of graft rejection.^[^ ^67^, ^68^, ^69^, ^70^, ^71^, ^72^ ^]^ Our study shows a critical role for BATF in regulating and stabilizing *Foxp3* expression, thus helps to maintain Treg identity and lineage stability in both lymphoid and non‐lymphoid tissues. Understanding the role of BATF in Treg maintenance and stability is a prerequisite to the design of strategies that enhance Treg function and therapeutic efficacy in autoimmune diseases and organ transplantation.

## 4 Experimental Section

### Mice

Treg‐specific BATF KO mice were generated by breeding *Foxp3* ^YFP‐Cre^ mice, a generous gift from Dr. Calvin Williams (016959, Jackson Laboratory), and BATF^F/F^ mice, which were kindly provided by Dr. E. Taparowsky.^[^ ^40^ ^]^ Treg‐specific BATF KO lineage tracer mice (TBKLT) were generated by breeding mice Foxp3^YFP‐Cre^ BATF^F/F^ mice with Rosa^mT/mG^ mice (007576, Jackson Laboratory). All mouse handling was performed according to the requirements of the Institutional Animal Care and Use Committee (IACUC) Guidelines of the Medical College of Wisconsin (MCW) under protocol number AUA3003.

### Flow Cytometry

Data for flow‐cytometry‐based analysis were acquired using an LSR II (BD Biosciences) and an LSRFortessa X‐20 (BD Biosciences) cytometer. Cells from all tissues were processed using 10% RPMI cell media. For secondary lymphoid tissues like spleen, iLN, and thymus, cells were processed into a single cell suspension by mashing the organ against a 100 µm filter, and then erythrocytes were lysed using ACK lysis buffer (A1049201, ThermoFisher). Cell processing from lung tissue involved first mincing the tissue into small pieces, followed by treatment with collagenase IV (1 mg mL^−1^) at 37 °C for 30 min. Subsequent processing was performed in a manner like that of secondary lymphoid organs. Hepatic lymphocytes were isolated after processing tissue into a single cell suspension using a 30% Percoll (Sigma‐Aldrich) solution followed by centrifugation at 1700 rpm for 10 min without brake. Colons were harvested, adipose and Peyer's patch tissues removed, and cut longitudinally. Next, colonic tissues were washed with cold PBS and minced into pieces. To remove the epithelial layers, the small pieces of tissues were dissolved in 5 mM EDTA (Lonza) Ca^2+^‐ and Mg^2+^‐ free RPMI 1640 medium (Life Technologies) and incubated at 37 °C for 30 min while shaking. This tissue was then further cut into fine pieces and digested with Collagenase II and III (1 mg mL^−1^; Worthington) and DNase I (200 µg mL^−1^; Roche) while incubating at 37 °C for 40 min. Lymphocytes were then isolated with 30%−60% Percoll gradient and washed with cold DPBS. All fluorophore‐labeled antibodies were purchased from BioLegend. Transcription factor staining including FOXP3 was performed using the True Nuclear Transcription Factor Buffer Kit (BioLegend). Herein, Tregs were described as “Foxp3+ Treg” only when they were identified by direct staining for FOXP3 by fluorophore‐conjugated antibody. Intracellular cytokine staining was performed using the protocol from BioLegend after in vitro stimulation of the cells with 50 ng mL^−1^ phorbol 12‐myristate 13‐acetate (Sigma‐Aldrich) and 1 µM ionomycin (Sigma‐Aldrich) in 10% RPMI for 30 min at 37 °C. To block Golgi apparatus transport, Brefeldin A (2 mg mL^−1^. BioLegend) was then added for 4.5 h. Flow cytometry data were analyzed using FlowJo V10 (BD Biosciences) software.

### Histology

Histological analysis of different tissues was performed using H&E staining on paraffin‐embedded tissue section with the assistance of the Children's Research Institute Histology Core. The analysis was performed using a Motic slide scanner and Motic DSAssistant software using 20× magnification.

### scRNA‐seq and Analysis

For scRNA‐seq experiments, GFP^+^ Tregs were sorted from the spleen, mLN, and colon of WLT and TBKLT mice at 7 weeks of age using a BD‐FACS‐Melody Sorter (BD Biosciences). Sorted cells were loaded onto the 10x Genomics Chromium Controller with a target cell number of 10,000 cells per mouse. Cells from all three tissues per mouse were loaded together and hashed using the HTO‐10x protocol.^[^ ^73^ ^]^ The libraries were prepared using the Chromium Single Cell v3 Reagent Kit (10x Genomics). Quantification was performed using a Kapa Library Quantification Kit (Roche Sequencing) and then was loaded onto an Illumina NextSeq 500 sequencer with the NextSeq 500/550 High Output Kit v2 (150 cycles; FC‐404‐2002; Illumina) with the following conditions: 26 cycles for read 1, 98 cycles for read 2, and 8 cycles for the i7 index read. Python Run Downloader (Illumina) was used to download raw sequencing data, which was then demultiplexed and converted to gene‐barcode matrices using the Cellranger (version 4.0) mkfastq and count functions, respectively (10× Genomics). All downstream analysis was performed in R (version 3.6.0) using the package Seurat (version 2.3.3).^[^ ^74^ ^]^ During the analysis, cell cycle score was calculated for all cells and regressed out.

### In Vitro Treg Culture

For iTreg generation, in vitro cells were enriched for naïve CD4 T cells using the Mojosort naïve CD4 T cell isolation kit (480039, BioLegend). Wells were coated with anti‐mouse CD3 antibody (0.5 µg mL^−1^) for 2 h. One million cells were seeded per well with anti‐mouse CD28 antibody (1 µg mL^−1^) and TGFβ (5 mg mL^−1^) in 10% RPMI on day 0. The following day, human IL‐2 was added to culture (100 U mL^−1^). On day 3, cells were washed and split onward with IL‐2. For tamoxifen treatment, 4‐OH tamoxifen (H7904‐5MG, Sigma; stock 5 mg mL^−1^) was added at a concentration of 1 µM or 2 µM to the in vitro cultured sorted Tregs. PBS was added for the control. The cells were kept in culture with anti‐mouse CD3 antibody (0.5 µg mL^−1^), anti‐mouse CD28 antibody (1 µg mL^−1^), and human IL‐2 (100 U mL^−1^).

### ATAC‐seq

ATAC‐seq (Assay for transposase accessible chromatin with sequencing) was performed using sorted CD4^+^ GFP^+^ T cells from spleens of TBKLT mice and WLT control mice using a BD FACSAria II sorter. Three independent experiments were performed using mice between 5 and 7 weeks of age. Cells were first enriched for CD4 T cells using the EasySep Mouse CD4 T cell isolation kit (19852, STEMCELL) and stained with anti‐mouse CD4 PE‐Cy7 antibody in 10% FBS, 2 mM EDTA in PBS. Cellular processing was done using 50 000 sorted CD4^+^ GFP^+^ T cells from each mouse following published ATAC‐seq protocols.^[^ ^75^ ^]^ Sequencing of barcoded amplified accessible regions were performed using an Illumina Nextseq 550 platform with 37 cycles of paired‐end sequencing and 40 to 50 million reads per sample.

### ATAC‐seq Analysis

Reads were first aligned to the *Mus musculus* mm10 genome using Bowtie2. Reads that were unpaired, unmapped, not primarily aligned, having low MAPQ values, or PCR duplicates were filtered out using SAMtools (Version 1.9).^[^ ^76^ ^]^ At the same time, reads aligning with the mitochondrial genome were removed as well. To call the peaks, MACS2 was used (–no model –shift −100 –ext size 200 ‐q 0.01) (version 2.1.1).^[^ ^77^ ^]^ Data from different samples were analyzed with DiffBind (version 2.12.0); only peaks identified within both replicates were used. Regions with differential accessibility were identified with DESeq2 (version 1.24.0)^[^ ^78^ ^]^ within the DiffBind analysis (version 2.12.0) and Manorm (Version 1.1.4).^[^ ^79^ ^]^ HOMER (version 4.9.1) was used for motif analysis. Gene ontology analysis of subset‐specific enhancers was done using GREAT (version 3.0.0).^[^ ^80^ ^]^

### ChIP‐seq Analysis

BATF ChIP‐seq data in Tregs was analyzed using published data.^[^ ^53^ ^]^ Raw fastq files were downloaded using SRAtools. The processing of the data was performed in the same way as ATAC‐seq data, mentioned above.

### Methylation Sequencing and Analysis

FACS‐sorted GFP^+^ Tregs from spleens of male WLT and TBKLT mice at 7 weeks of age were used for methylation analysis. DNA was extracted using Dneasy Blood and Tissue Kit (69504, Qiagen) followed by bisulfite treatment using EpiTect Bisulfite Kit (59104 Qiagen). Treated DNA was used for amplification targeting the CNS2 region of the *Foxp3* locus using the following primers: CNS2 CpG 1–6, TGGGTTTTTTTGGTATTTAAGAAAGAT and 5′Biosg/AAAAAACAAATAATCTACCCCACAA; CNS2 CpG 7–10, 5′Biosg/GGGTTTTGTATGGTAGTTAGATGG and ACAACCTAAACTTAACCAAATTTTTCTAC. Sequencing was performed using Pyromark q48 reagents following the manual.

### Statistical Analysis

Statistical tests were performed using GraphPad Prism 7. *p* values were calculated using an unpaired t‐test using a parametric test for Gaussian distribution. A *p*‐value < 0.05 was considered to indicate statistical significance (ns = not significant, *p < 0.05, ^**^ *p* < 0.01, and ^***^ *p* < 0.001).

## Conflict of Interest

The authors declare no conflict of interest.

## Author Contributions

A.K. and X.W. contributed equally to this work. Achia Khatun was responsible for designing and performing experiments and writing the manuscript. X.W. performed experiments with cellular immunology, analyzed the data, and edited the manuscript. F.Q. contributed to the methylation sequencing and analysis. L.F. helped with the histology analysis. A.C. helped with designing scRNA‐seq experiments. M.R.K. assisted with writing, reviewing, and editing the manuscript. M.Y.K. edited the manuscript. A.K. helped with in vitro experiments. R. B. contributed to bioinformatic analysis. Y.G.C. contributed to the experimental design. N.S. helped with data analysis. C.B.W. contributed to experimental design, data analysis, and manuscript editing. W.C. guided designing experiments revised and edited the manuscript, and supervised the study.

## Supporting information

Supporting Information

## Data Availability

The data that support the findings of this study are available from the corresponding author upon reasonable request.
