# Supplementary material for: BATF is Required for Treg Homeostasis and Stability to Prevent Autoimmune Pathology
Source: Adv Sci (Weinh). 2023 Aug 16;10(28):2206692. doi: 10.1002/advs.202206692 (PMC10558681; doi:10.1002/advs.202206692)
Supplement: Supplementary file 1 — Supporting Information [file ADVS-10-2206692-s001.pdf]

ADVANCED SCIENCE
Open Access

Supporting Information

for *Adv. Sci.*, DOI 10.1002/advs.202206692

BATF is Required for Treg Homeostasis and Stability to Prevent Autoimmune Pathology

Achia Khatun, Xiaopeng Wu, Fu Qi, Kexin Gai, Arjun Kharel, Matthew R. Kudek, Lisa Fraser, Ashley Ceicko, Moujtaba Y. Kasmani, Amber Majnik, Robert Burns, Yi-Guang Chen, Nita Salzman, Elizabeth J. Taparowsky, Dayu Fang, Calvin B. Williams and Weiguo Cui**

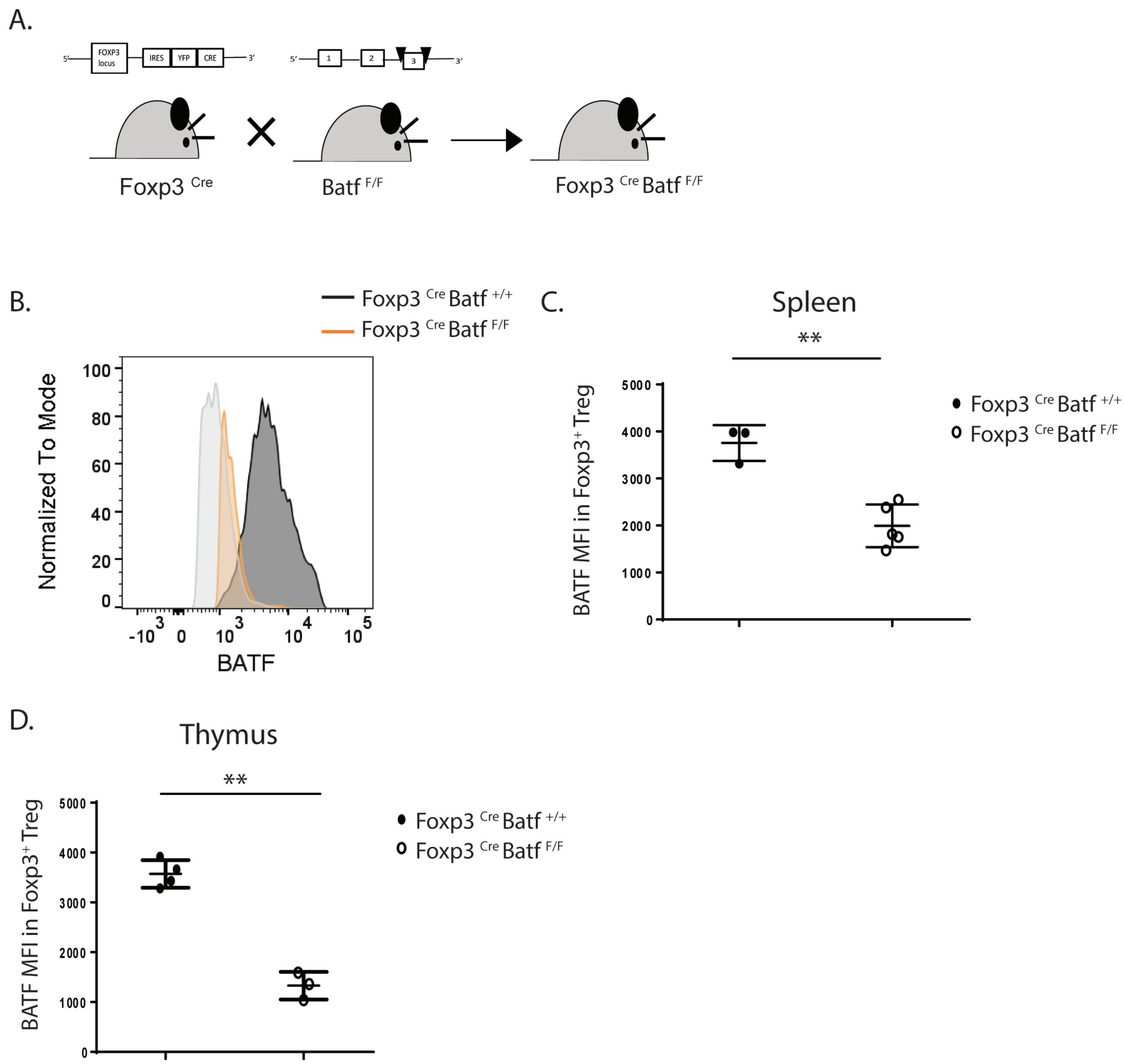

Figure S1. Development of Treg sp BATF KO mice and validation. Related to Figure 1.

A. Schematic representation of generating Treg specific BATF KO mice (Foxp3Cre BatfF/F). **B-D.** Representative histogram (B) and quantified dot plot showing expression of BATF in FOXP3+ Tregs in spleen (C) and thymus (D) of Foxp3Cre BatfF/F and control (Foxp3Cre Batf+/+) mice around 5 to 10 weeks of age. The data represented here were pulled from three individual repeats with 2-5 mice per group. Data here are shown ± SEM. *P < 0.05, **P < 0.01, ***P < 0.001 (two tailed unpaired t test)

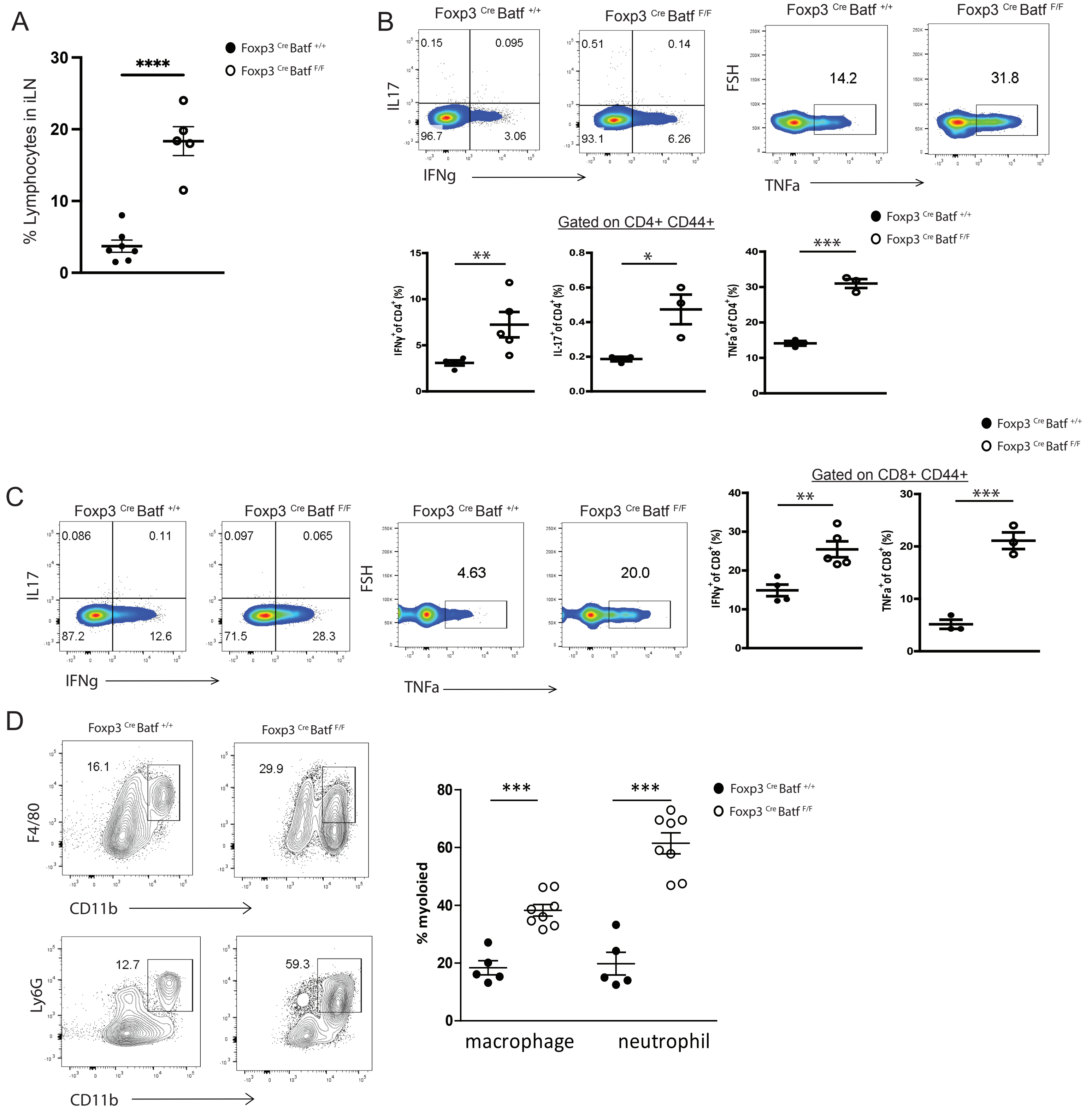

Figure S2. BATF in Tregs is required for homeostatic regulation involving both adaptive and innate immunity. Related to Figure 1.

A. Dot plots showing the frequency of lymphocytes in the inguinal lymph nodes from Foxp3Cre BatfF/F mice compared to control Foxp3Cre Batf+/+ mice. **B.** Representative flow plots (left) and quantified dot plots (right) showing frequency of activated CD4 T cells producing IFNγ, IL17, and TNFα in Foxp3Cre BatfF/F mice compared to control (Foxp3Cre Batf+/+) mice. **C.** Representative flow plots (left) and quantified dot plots (right) showing frequency of activated CD8 T cells producing IFNγ and TNFα in Foxp3Cre BatfF/F mice compared to control mice. **D.** Representative flow plots (left) and quantified dot plots (right) showing frequency of innate immune cells macrophages and neutrophils in Foxp3Cre BatfF/F mice compared to control mice. These data were from mice around 8 to 11 weeks of age. All data are pooled from at least 3 individual repeats with a minimum of 3-5 mice per group. Data here are shown ± SEM. *P < 0.05, **P < 0.01, ***P < 0.001 (two tailed unpaired t test)

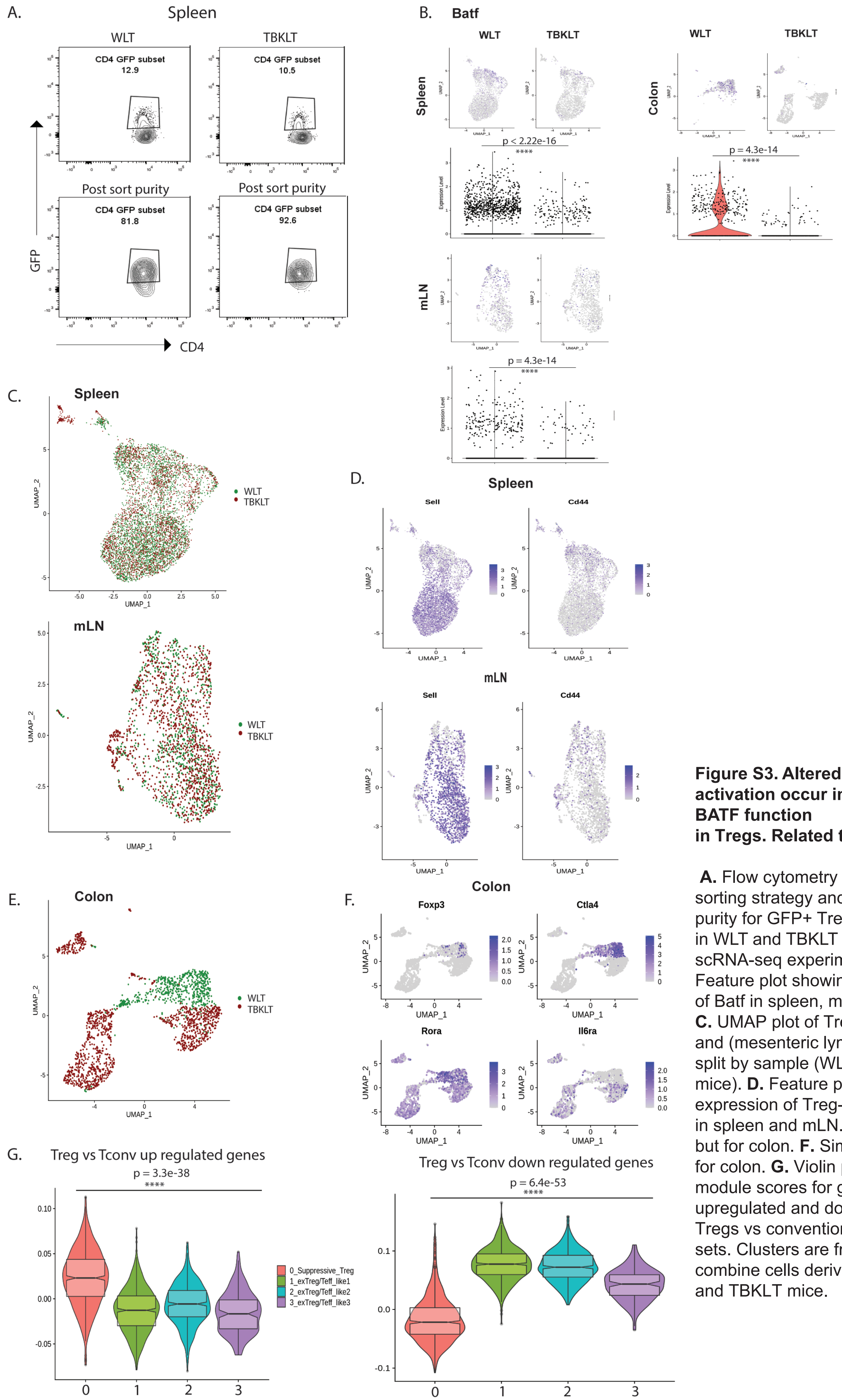

Figure S3. Altered Treg fate and activation occur in absence of BATF function in Tregs. Related to Figure 2.

A. Flow cytometry plot showing sorting strategy and post-sort purity for GFP+ Tregs from spleen in WLT and TBKLT mice used for scRNA-seq experiments. **B.** Feature plot showing expression of Batf in spleen, mLN, and colon. **C.** UMAP plot of Tregs in spleen and (mesenteric lymph node) mLN split by sample (WLT and TBKLT mice). **D.** Feature plot showing expression of Treg-specific genes in spleen and mLN. **E.** similar to C, but for colon. **F.** Similar to D, but for colon. **G.** Violin plots showing module scores for gene sets upregulated and downregulated in Tregs vs conventional T cells gene sets. Clusters are from colon and combine cells derived from WLT and TBKLT mice.

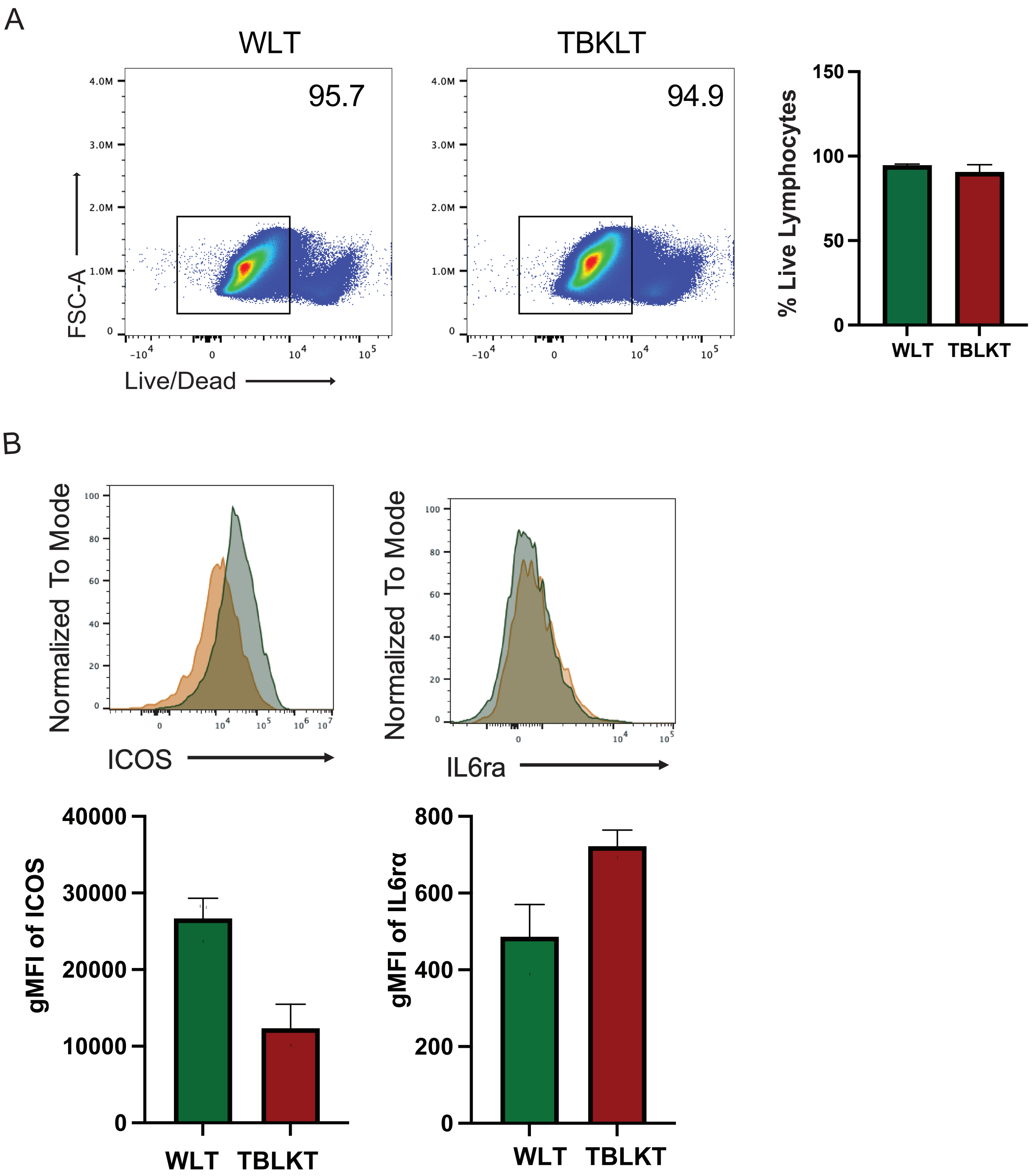

Figure S4. Loss of BATF promotes exTreg phenotype in large intestine. Related to Figure 3.

A. Flow cytometry plot (left) and bar plot (right) showing viability of total lymphocytes from large intestine of WLT and TBKLT mice **B.** Flow cytometry plot (up) and bar plot (down) showing per cell expression (gMFI) of ICOS and IL6ra in Foxp3^{+} Tregs from large intestine of WLT and TBKLT mice. Foxp3^{+} expression in Tregs is conferred by direct staining with and detection of intranuclear fluorophore conjugated antibody. These data were from male mice around 9 weeks of age. All data are pooled from at least one experiment with a minimum of 2-3 mice per group. Data here are shown ± SEM.

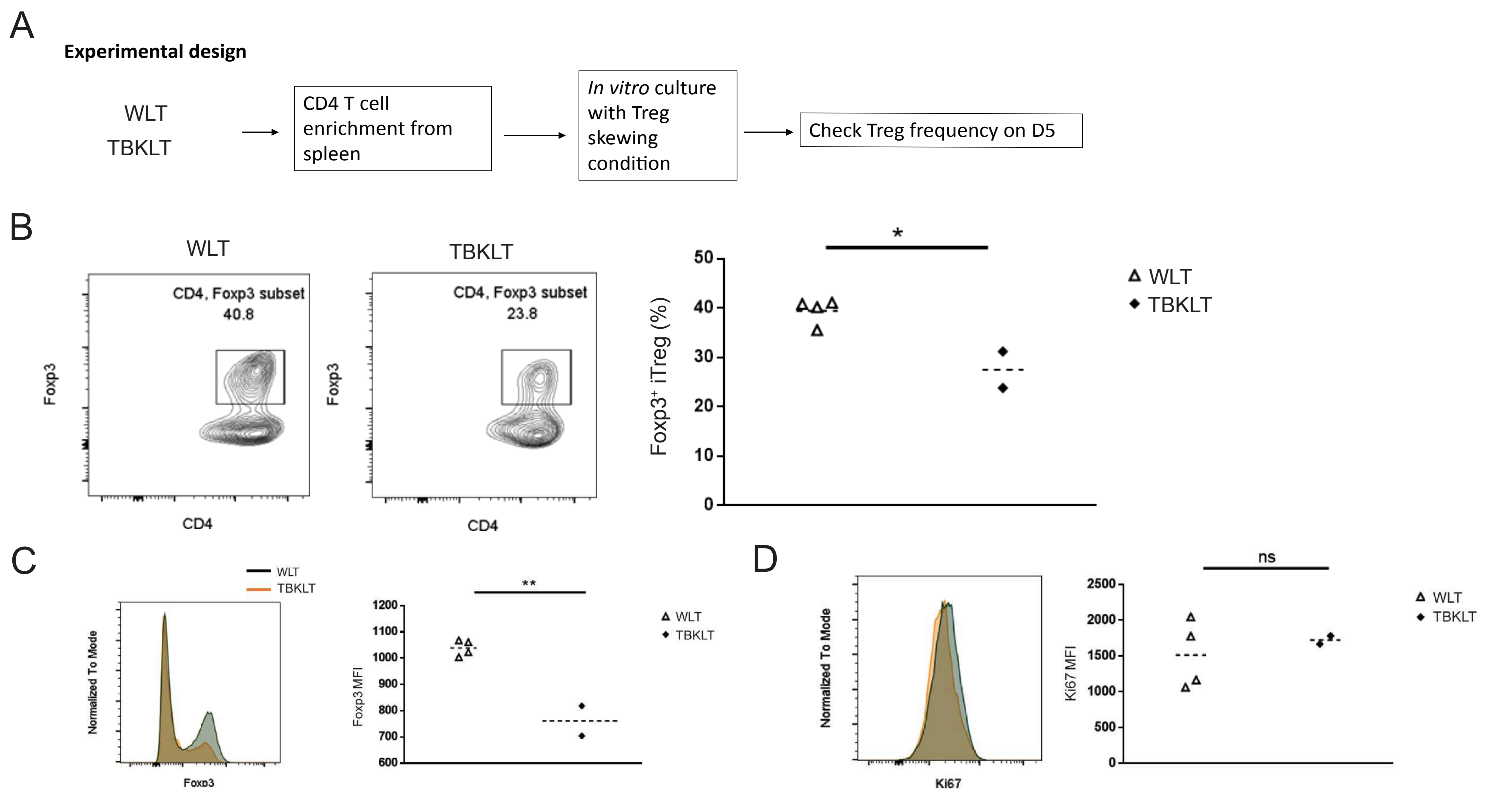

Figure S5. BATF is needed for iTreg differentiation. Related to Figure 3.

A. Experimental design for *in vitro* iTreg differentiation using naïve CD4 T cells from spleens of WLT and TBKLT mice. **B.** Frequency of Foxp3+ Tregs in CD4 T cells from WLT and TBKLT mice cultured for 5 days in Treg skewing conditions. **C and D.** Representative histograms (left) and quantified dot plots (right) showing expression of FOXP3 (C) and Ki67 (D) in FOXP3+ Tregs. The data represented here were pulled from two individual repeats (A-D) with 1-3 mice per group. Data here are shown ± SEM. *P < 0.05, **P < 0.01, ***P < 0.001 (two tailed unpaired t test).

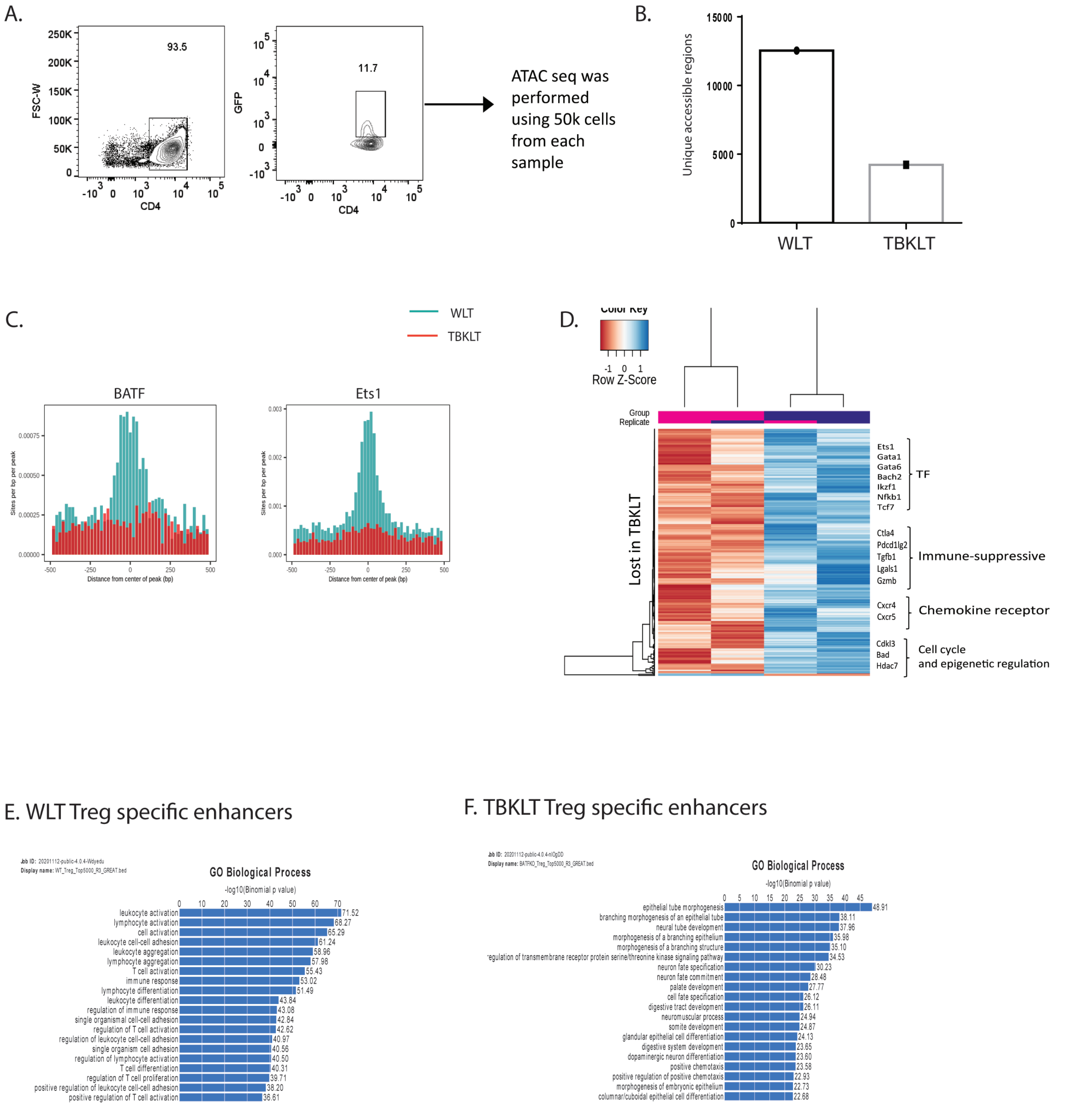

Figure S6. **Accessibility of Treg-specific genes is regulated by BATF in Tregs. Related to Figure 5.**

A. Sorting strategy for Tregs used for ATAC-seq. **B.** Bar plot showing the number of uniquely accessible enhancer regions in TBKLT mice compared to WLT control mice. **C.** Histogram showing the enrichment of BATF and Ets1 binding motifs in WLT Treg specific enhancers compared to enhancers in TBKLT mice. **D.** Heatmap showing enhancer regions with differential accessibility in WLT and TBKLT mice analyzed using DESeq2 from the DiffBind package. Select genes putatively associated with these differentially accessible enhancer regions are labeled. **E-F.** Bar plot showing pathways associated with enhancer regions uniquely accessible in either WLT (left) or TBKLT (right) mice, analyzed by GREAT. Data are from two independent experiments performed using TBKLT mice and WT control at 7 weeks of age.

A.

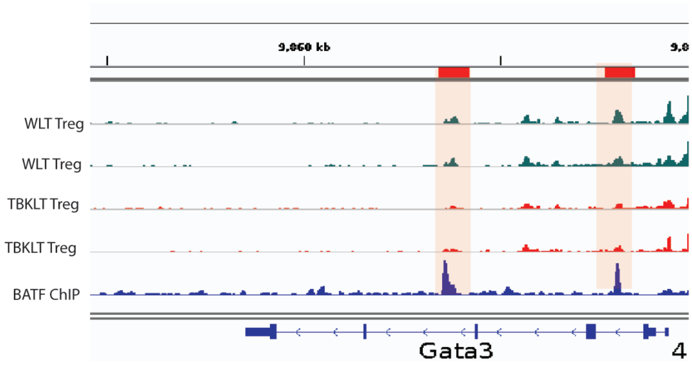

B.

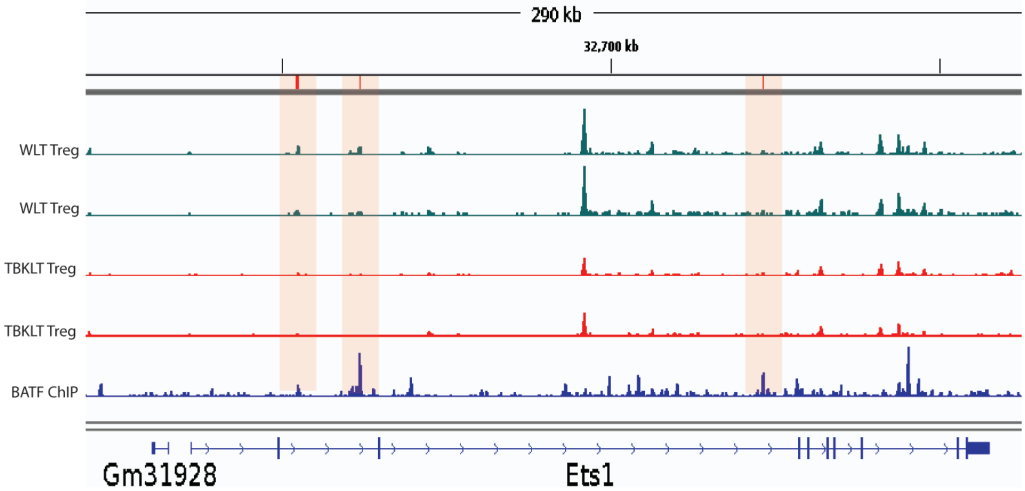

Figure S7. **BATF regulated accessibility of genes involved in Treg stability. Related to Figure 5.**

Representative tracks of WLT and TBKLT Treg ATAC-seq data and published Treg BATF ChIP-seq data in the enhancer regions of Gata3 (A) and Ets1 (B).

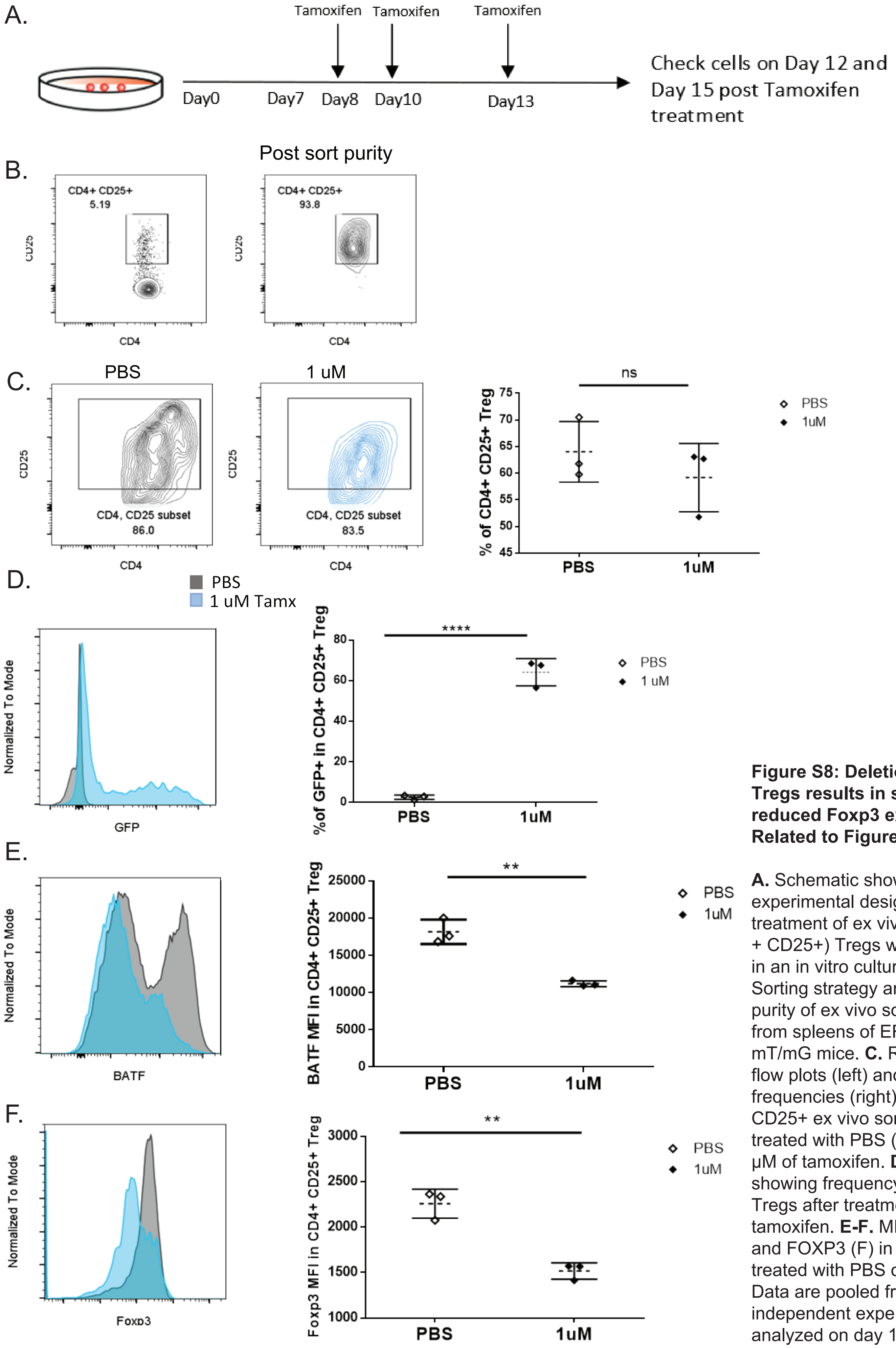

Figure S8: Deletion of BATF in Tregs results in significantly reduced Foxp3 expression. Related to Figure 6.

A. Schematic showing experimental design for the treatment of ex vivo sorted (CD4 + CD25+) Tregs with tamoxifen in an in vitro culture system. **B.** Sorting strategy and post sorting purity of ex vivo sorted Tregs from spleens of ERCreBatfF/F mT/mG mice. **C.** Representative flow plots (left) and quantified frequencies (right) of CD4+ CD25+ ex vivo sorted Tregs treated with PBS (control) or 1 μM of tamoxifen. **D.** Similar to C, showing frequency of GFP+ Tregs after treatment with PBS or tamoxifen. **E-F.** MFI of BATF (E) and FOXP3 (F) in ex vivo Tregs treated with PBS or tamoxifen. Data are pooled from two independent experiments analyzed on day 12 or day 15 post-treatment with 1-2 mice per group. Data here are shown ± SEM. *P < 0.05, **P < 0.01, ***P < 0.001 (two tailed unpaired t test).
